# Correlation of methylation status in MTHFR promoter region with recurrent pregnancy loss

**DOI:** 10.1186/s43141-021-00147-w

**Published:** 2021-03-22

**Authors:** Mai Mahmoud Shaker, Taghreed Abdelmoniem shalabi, Khalda said Amr

**Affiliations:** 1 Prenatal and Fetal Medicine Department, Human Genetics and Genome Research Division, National Research Centre, 33 El Buhooth St, Dokki, Cairo, Egypt; 2 Medical Molecular Genetics Department, Human Genetics and Genome Research Division, National Research Centre, 33 El Buhooth St, Dokki, Cairo, Egypt

**Keywords:** Pregnancy loss, Methylation, Methylenetetrahydrofolate reductase, Polymorphism, Thrombophilia

## Abstract

**Background:**

DNA methylation is an epigenetic process for modifying transcription factors in various genes. Methylenetetrahydrofolate reductase (*MTHFR*) stimulates synthesis of methyl radical in the homocysteine cycle and delivers methyl groups needed in DNA methylation. Furthermore, numerous studies have linked gene polymorphisms of this enzyme with a larger risk of recurrent pregnancy loss (RPL), yet scarce information is available concerning the association between epigenetic deviations in this gene and RPL. Hypermethylation at precise DNA sequences can function as biomarkers for a diversity of diseases. We aimed by this study to evaluate the methylation status of the promoter region of *MTHFR* gene in women with RPL compared to healthy fertile women. It is a case–control study. Hundred RPL patients and hundred healthy fertile women with no history of RPL as controls were recruited. *MTHFR* C677T was assessed by polymerase chain reaction-restriction fragment length polymorphism (RFLP). Quantitative evaluation of DNA methylation was performed by high-resolution melt analysis by real-time PCR.

**Results:**

The median of percentage of *MTHFR* promoter methylation in RPL cases was 6.45 [0.74–100] vs. controls was 4.50 [0.60–91.7], *P* value < 0.001. In the case group, 57 hypermethylated and 43 normo-methylated among RPL patients vs. 40 hypermethylated and 60 normo-methylated among controls, *P*< 0.005. Frequency of T allele in C677T *MTHFR* gene among RPL patients was 29% vs. 23% among the control group; C allele vs. T allele: odds ratio (OR) = 1.367 (95% confidence interval (CI) 0.725–2.581).

**Conclusion:**

Findings suggested a significant association between hypermethylation of the MTHFR promoter region in RPL patients compared to healthy fertile women.

## Background

RPL is defined as more than two to three consecutive pregnancy losses earlier to the 20th week of gestation [1]. Around 1–5% of married couples suffer from this stressful event worldwide. Full-term pregnancies embraces mechanisms that avoid expulsion of fetus semi-allograft as well as sharp regulation measures for fluctuations in maternal environment in response to any changes required during fetal growth. Epigenetic mechanism involving DNA methylation has a great impact on monitoring the evolution processes of the trophoblastic cell lines [2, 3]. Changes in carbon metabolic pathway which is the main provider of one carbon units are expected to affect methylation arrangements [4].

In early gestation weeks during initial steps of fetal growth formation, DNA methylation of fetal germ cells is needed for regular spermatogenesis and fetal growth after conception [5]. The keys in epigenetic mechanisms are DNA methylation, post-translational histone modifications, chromatin remodeling, and regulation of small noncoding RNA [6]. Still DNA methylation is the utmost commonly assessed epigenetic modification in context of RPL. It represents the methylation of cytosines at CpG (cytosine–phosphate–guanine) dinucleotide. DNA methylation regularly happens at CpG islands in promoter region of the genes to adjust gene expression, Though hypermethylation of DNA in 5′-CpG-3′ islands is related with gene suppression, hypomethylation among those regions is related to transcriptional activation [7]. Lucas et al. [8] investigated how alteration in methylation at the level of stem cell methylation can be responsible for active variations in stem cell populations which leads to abnormal plasticity of the uterine endometrium and consequently pregnancy loss. Pliushch et al. [9] stated that epimutations that lead to unsuitable methylation and expression patterns of genes may add to increased incidence of pregnancy failures.

One of the common thrombophilia genes is *MTHFR* gene. It is essential in making methyl groups needed for DNA methylation, repair, and synthesis. During gestation period, females experience lots of physiological changes; these changes influenced by methylation pattern of some genes. *MTHFR* have a critical role during gestation period due to its enrollment in some thrombotic events and methylation process. Hypermethylation of the promoter region of *MTHFR* gene decreases gene expression. Consequently, it lowers the accessibility of methyl groups needed for entire DNA methylation [10]. DNA methylation leads to the addition of a methyl group at 5′ carbon of cytosine which can bring changes in DNA structure as well as altering the set patterns of gene expression. Moreover, *MTHFR* C677T polymorphism may lead to decrease in enzymatic activity hindering transmethylation cycle which in return diminish the availability of free methyl groups and slowing down DNA methylation process [11]. We believe that in cases of RPL especially (unexplained RPL), early finding of new prognostic marker would be of great help to obstetricians for early detection and management of RPL patients. We aimed by this study to evaluate the methylation status of the promoter region of *MTHFR* gene in RPL patients compared to healthy fertile women.

## Methods

### Recruitment of participants

This is a case–control study. Participants included in the two studied groups had a comparable age 25.7±5.1years vs. 26.2±4.7years, *p* = 0.495 and body mass index (BMI) 26.4±2.5 vs. 27.0±2.7, *p* = 0.140. Participants were referred to our Recurrent Pregnancy Loss clinic at our institute, from the period of September 2018 to March 2020. Our inclusion criteria for the case group included hundred RPL patients who had passed through at least two to three consecutive pregnancy losses with the same spouse. None of the participants were smokers or alcoholic. Patients with recurrent pregnancy loss risk factors like anatomic malformations, karyotype abnormality, disturbance in hormonal levels mainly (thyroid profile and progesterone), immunological abnormalities (anticardiolipin antibodies, anti-β2 glycoprotein 1 antibody, antithyroglobulin antibody and antinuclear antibody), history of thrombotic events, inherited thrombophilia mutations, deficiency for protein c and s, and infections (cytomegalovirus, rubella, and chlamydia trachomatis) were excluded. Hundred healthy fertile women were recruited in the control group who had full-term pregnancy, gave birth to healthy children, and have not experienced any form of pregnancy loss or any pregnancy-related complications. All participants signed a written informed consent before sampling and the study was accepted by the Medical Ethical Committee of NRC.

### Extraction of genomic DNA

After blood sampling, 2 mL of whole blood was collected into Ethylenediaminetetraacetic acid (EDTA)-containing tubes for genomic DNA extraction. Genomic DNA was isolated from whole blood using salting out technique [12]. DNA was eluted in 100 μl of distilled water and finally stored at −20°C till analysis.

### Genotyping of C677T polymorphism in MTFHR gene

Genotyping for C677T SNP was analyzed using PCR and restriction fragment length polymorphism analysis (RFLP). The sequence of primers used was as follows: forward primer 5′-TGTGGTCTCTTCATCCCTCGC-3′ and reverse primer 5′-CCTTTTGGTGATGCTTGTTGGC-3′ [13]. PCR thermal condition was as follows: initial denaturation for 5 min at 95 °C, 30 cycles of 30s at 94 °C, annealing at 60°C for 30s and extension for 30s at 72°C and a final extension for 10 min at 72°C. Two-microliter PCR yields were loaded in 3% agarose gel to check for successful amplification, band size (513 bp). Electrophoresis was done. All PCR products were allied against a 100bp DNA Ladder (Fermentas, Germany).

**Fig. 1:**
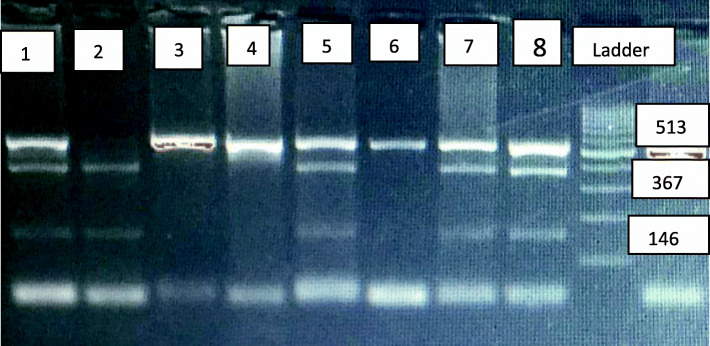
Digested pcr products using Hinf 1 enzyme for C667T polymorphism. 3% agrose gel showing digested pcr products: (a) lanes 1, 5, 7, and 8 showing 513,367 and 146 bp bands (CT genotype); (b) lane (2) showing 367 and 146 bp bands (TT genotype); (c) lanes 3, 4, and 6 showing 513 bp band (CC genotype); and (d) ladder, 100 base pair (bp) molecular marker

Fast Digest HinfI (Thermo Scientific; Thermo Fisher Scientific) was used for the RFLP step. Final mixture reaction of total volume of 15 μl was prepared with 1 μl of HinfI enzyme, 1.5 μl Buffer with a concentration of (10×), 2.5 μl of sterile H_2_O, and 10ng of PCR final product. The reaction was then incubated at 37°C for 35 min in a thermal cycler then followed by inactivation step by heating at 80°C for 25 min. If *MTHFR* C677T polymorphism is found, the exchange of C with T generates a restriction site for Hinf I and the PCR product of 513 bp is cut in two pieces, one of 146 bp and the other of 367 bp [13] as shown in Fig. 1.

### Bisulphite DNA conversion

Five hundred nanograms of purified genomic DNA was bisulfite-treated using the EZ DNA Methylation ™ Kit (Cat No. D5006, Zymo Research, CA, USA). The bisulfite-modified DNA was used for gene-specific methylation analysis of Promoter region of MTHFR gene. In each run, positive control (100% methylated) and negative control (0% methylated) were used using Methylated and Unmethylated DNA CpG Genome (Chemicon International Inc.) as a reference.

### Real-time methylation quantification analysis

**Fig. 2:**
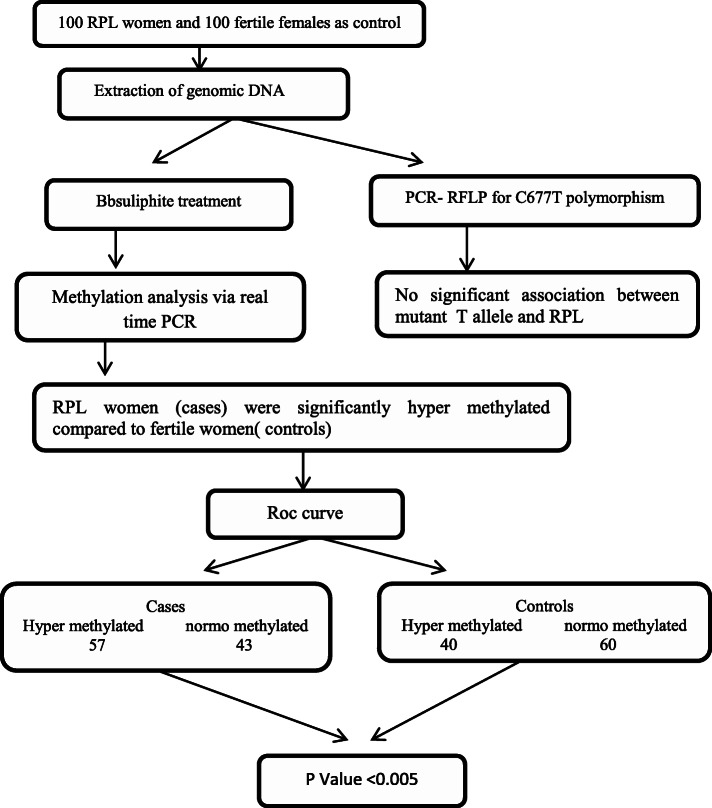
Study plan and outcome

Real-time PCR reactions was performed following this amplification protocol: 15 min on 95°C, 52 cycles of 32s on 95°C, 30s at 57°C, 30 s at 72°C with a last extension lead of 35 min at 72 °C. The following are the methylated primers used: frontward 5-TTAGGAGTGGTTGTAGACGGGT-3 and reverse 5-GAATAACTCAAAACGCTCGAC-3. The unmethylated primers were: frontward 5-TTAGGAGTGGTTGTAGATGGGT-3 and reverse 5-CCCAAATAACTCAAAACACTCAAC-3. The primers were designed precisely for methylated and unmethylated regions in the first CpG Island at *MTHFR* coding region with comparable annealing temperatures. To assess the percentage of methylation status, every single sample was normalized to a non-CpG promoter region of the *MTHFR* gene. 2ΔΔCt equation was used to resolute the relative degree of methylation [14]. The overall study plan and results outcome is summarized in brief in Fig. 2.

### Statistical analysis

**Fig. 3:**
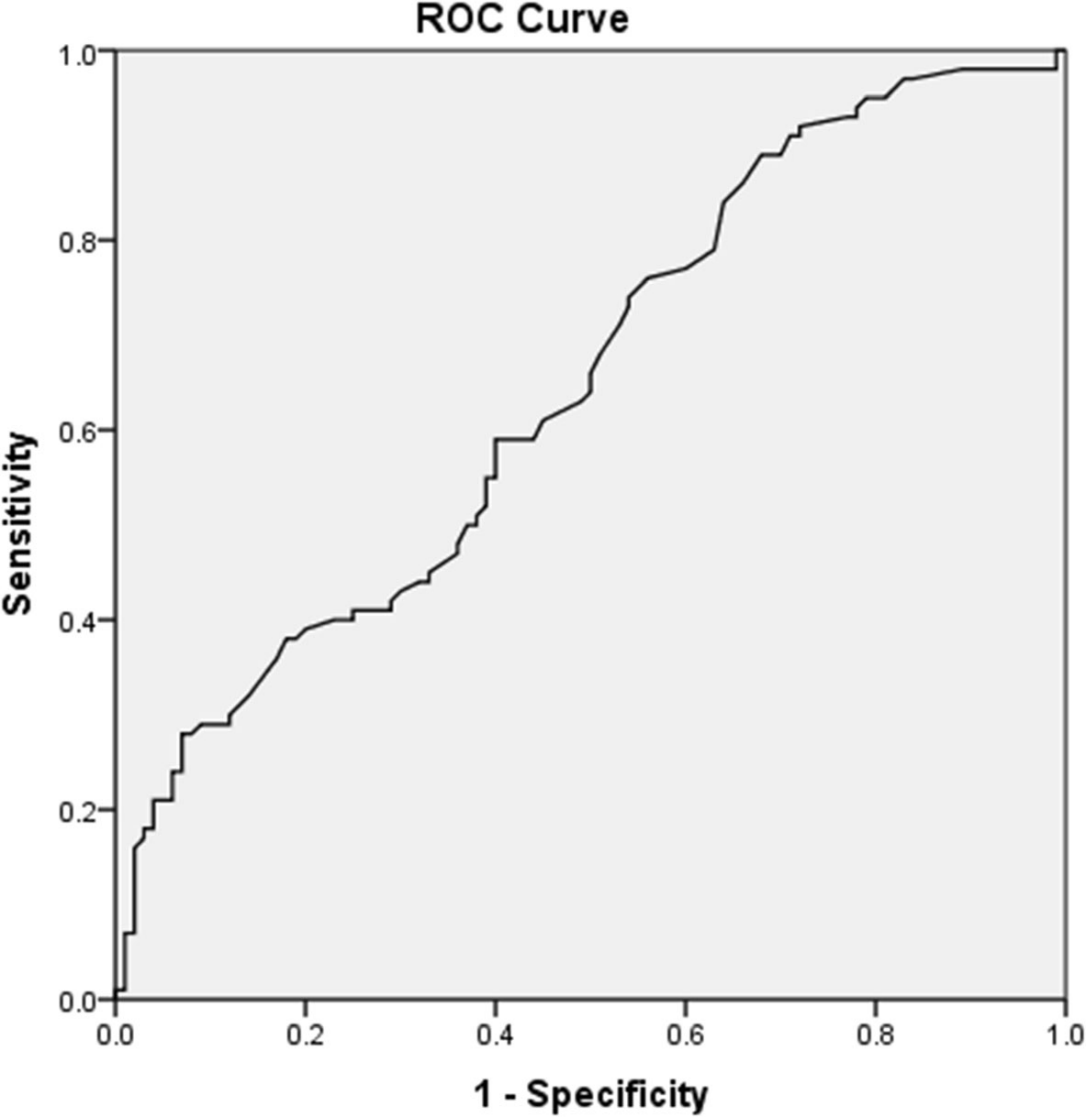
Roc curve analysis of methylation profile of gene-specific MTHFR. AUC, area under the curve; 95% CI, 95% confidence interval

Statistical analysis was done using IBM© SPSS© Statistics version 22 (IBM© Corp., Armonk, NY, USA). Numerical data were expressed as mean and standard deviation or median and range as appropriate. Qualitative data were expressed as frequency and percentage. Fisher’s exact test was used to examine the relation between qualitative variables. For quantitative data, comparison between two groups was done using independent sample *t*-test. Logistic regression was used to assess the risk of genotypes and alleles. The odds ratio (OR) with it 95% confidence interval (CI) were used for risk estimation. A receiver operating characteristic (Roc curve) was done to obtain a cuff off value for the methylation percentage in order to categorize participants to either normo- or hypermethylated and *p* value was obtained using chi-square test. A *p* value < 0.05 was considered significant (Fig. 3).

## Results

The descriptive parameters (age, BMI, and consanguinity rate) between the two studied groups showed no significant difference observed that needed to be highlighted later on in the “Discussion” section (26.2 ± 4.7, 27.0 ± 2.7,48 %) vs. control (25.7 ± 5.1, 26.4 ± 2.5, 36%); *P* values: 0.49, 0.14, 0.08 (Table 1).

**Table 1:** Descriptive characteristics among RPL patients and controls

		Cases (***n***=100)	Controls (***n***=100)	***P*** value
**Age (**mean ± SD**)**		26.2 ± 4.7	25.7 ± 5.1	0.49
**BMI (** mean ± SD**)**		27.0 ± 2.7	26.4 **±** 2.5	0.14
**Consanguinity**	Positive	48%	36%	
	Negative	52%	64%	0.08
**No. abortions**	\	4 ± 1.1	NA	
**Pregnancy loss**	1st trimester	28%	NA	
	2nd trimester	72%		
**Live births**	0	64	0	
	1	35	15	
	2	1	42	
	3	0	43	

*BMI* body mass index, *No. abortions* number of abortions, *NA* not available

The median of percentage of *MTHFR* promoter methylation in RPL cases was 6.45 (0.74–100) vs. controls was 4.50 (0.60–91.7); *P* value < 0.001 (Table 2). On evaluating roc curve for sensitivity, specificity, and area under the curve, a cutoff value of 5.5 as a threshold for methylation percentage was defined. Among RPL patients, 57 were hypermethylated and 43 were normo-methylated vs. 40 hypermethylated and 60 normo-methylated among the control group with a significant *P*< 0.005 (Table 3).

**Table 2:** Methylation among cases of pregnancy loss compared to controls

	Cases (***n***=100)	Controls (***n***=100)	***P*** value
**Methylation, median (range)** **Mann–Whitney** ***U***	6.45 (0.47–100)	4.50 (0.60–91.7)	< 0.001

The *p* value < .05 is significant

**Table 3:** Number of hypermethylated and normo-methylated RPL cases vs. number of hypermethylated and normo-methylated controls

	Cases (***n***=100)	Controls (***n***=100 )	***P*** value
**Hypermethylated**	57	40	0.016
**Normo-methylated**	43	60	

The *p* value < .05 is significant

Analysis for C677T polymorphism was done after methylation quantification to assess if there is a direct correlation between the polymorphism and methylation status in coding region of *MTHFR* in RPL patients. Accordingly, the frequency of C677T genotype *MTHFR* gene in RPL patients showed CC genotype 48%, CT 46%, and TT 6% vs. control group—CC genotype was 58%, CT 38%, and TT 4% (CC vs. CT: OR = 1.463 (0.823–2.600); CC vs.TT: OR =1.812 (0.483–6.79); CC vs. CT+TT: OR= 1.496 (0.856–2.164); CT+TT vs. TT: OR= 1.532 (0.419–5.603)). There was no any significant difference in the TT genotype between the two groups. C allele in RPL patients was 71% vs. 77% in controls and T allele was 29% in RPL patients vs. 23% in control group. C allele vs. T allele OR: 1.367 (95% CI 0.725–2.581). Both groups were consistent with Hardy–Weinberg equation 0.242 in RPL patients and 0.466 in control group (Table 4).

**Table 4:** Frequencies of MTHFR gene polymorphic genotypes and alleles among cases of RPL compared to controls

	Patients (***n***=100)	Control (***n***=100)	OR	95%CI
**CC**	48 (48%)	58 (58%)	1	*Ref*
**CT**	46 (46.0%)	38% (38.0%)	1.463	(0.823–2.600)
**TT**	6 (6.0%)	4 (4.0%)	1.812	(0.483–6.796)
**CT+TT**	52 (52%.0)	42 (42.0%)	1.496	(0.856–2.614)
**CC+TT**	94 (94%)	96 (96.0%)	1	
**TT**	6 (6.0%)	4 (4.0%)	1.532	(0.419–5.603)
**C**	71 (71.0%)	77 (77.0%)	1	
**T**	29 (29.0%)	23 (23.0%)	1.367	(0.725–2.581)

The *p* value < .05 is significant

*OR* odds ratio, *CI* confidence intervals

On correlating specific methylation percentage with different genotypes among the whole studied population (cases and controls; *n*=200), the CC genotype showed significantly lower methylation compared to CT (*p* < 0.001) and TT genotypes (*p =* 0.027). CT and TT genotypes had comparable methylation (*p =* 1.000).

## Discussion

To maintain full-term pregnancy, it is important to have an adequate, controlled internal maternal environment for embryo’s growth and development. DNA methylation has a critical role in cellular growth and differentiation of trophoblastic cell lines [15]. The *MTHFR* gene which is the topic of our study is one of the non-imprinted genes involved in pregnancy loss. Hypermethylation of CpG islands is typically related with gene silencing. Epigenetic variations are hypothetically changeable. *MTHFR* gene is a thrombophilic bio-marker. It is located at the intersection of reactions generating methyl groups for regulating DNA methylation processes, repair, and synthesis. During gestational period, women go through main physiological and immunological changes mostly affected by methylation patterns of particular accountable genes [16]. *MTHFR* gene is one of several genes that have a significant role during gestation period by being involved in thrombotic actions or methylation processes. Hypermethylation in the promoter region of *MTHFR* gene is certain to decrease gene expression which in return lessen accessibility of methyl groups needed in global DNA methylation [10]. In addition, *MTHFR C677T* polymorphism causes a decrease in enzymatic activity affecting the transmethylation cycle and causing a hypo global DNA methylation due to lack of free methyl groups [11]. The authors aimed by this study to evaluate the methylation status of the promoter region of *MTHFR* gene in RPL patients in comparison to healthy fertile women.

The authors observed by looking at the results that frequency of methylation in *MTHFR* promoter region was significantly higher among RPL patients than in controls (*p* <0.05). We took a step further by doing receiver operating characteristic (ROC) assessment to find a threshold for methylation level; in this case, it was 5.5. To categorize the participants as either normo- or hypermethylated, ROC curve was plotted displaying true-positive rate (sensitivity) versus false-positive rate (one specificity) and area under the curve (AUC) = 0.647 and was used to assess how fit the estimate model could discriminate between the two groups (normo- and hypermethylated). We concluded that RPL patients were significantly higher regarding hypermethylation of the *MTHFR* promoter area compared to control group.

Several authors have strained to comprehend the role of *MTHFR C677T* polymorphism in correlation with the DNA hypermethylation; nevertheless, scarce studies did not find any association between the frequency of mutant allele and global DNA methylation [17]. According to our results, *MTHFR C677T* genotypic status of RPL patients in comparison to controls showed that there was an increase in the frequency of the CT and TT genotypes among RPL patients compared to the control group but did not reach statistical significance. Even when combining CT+TT genotypes against TT genotype, also no statistical significance was evident between RPL patients and controls. On allele level, C and T alleles had comparable frequencies between the two studied groups.

Due to scarcity of literature on *MTHFR* gene-specific methylation and RPL, the results are not validated with a substantial number of studies. However, comparable results were reached in a previous study which showed that *MTHFR* genotypic distribution among cases and controls had no significant difference *P*= 0.409 [18]. Then, the authors did another study few years later in north India and recruited 85 RPL patients and 121 fertile healthy controls and reported that women carrying methylated allele for *MTHFR* gene have a significant 3.6-fold increased risk for RPL. The amount risk of methylated allele in RPL women was found to be intensified from the normal genotype CC (2.8 folds) to CT (7.5 folds) individuals [19]. Mishra and his colleagues in 2019 published their study. The results showed that RPL cases were hypermethylated for *MTHFR* gene in comparison to healthy fertile female in the control group with a statistical significance *p*= 0.002. Hypermethylated RPL cases for *MTHFR* gene were 28 compared to72 age-matched controls. But in contrast to our finding, they reported that there was a positive correlation with C677T polymorphism. RPL patients carrying CT genotype had a significantly MTHFR gene-specific methylation *p=*0.005 [20]. Study done in Gutenberg University, Germany, had reported a very interesting finding. The study reported the DNA methylation patterns at two paternally methylated (*H19* and *MEG3*) and other four maternally methylated (*LIT1*, *NESP55*, *PEG3*, and *SNRPN*) imprinted areas among fetus muscle. Samples taken from aborts and stillbirths showed that 2 of 55 (4%) aborted fetuses and 10 of 57 (18%) stillbirths exhibited various imprinted genes with a hypermethylated pattern [9]. Furthermore Rotondo and his colleagues [21] reported that there was hypermethylation in promoter region of MTHFR in seminal samples of RPL couples and that CpG islands inside the promoter region were 100% methylated, i.e., hypermethylation of *MTHFR* promoter region. The study implicated that these different methylation patterns detected might possibly be related to inadequate maternal uterine environment leading to fetal loss in early pregnancy among RPL patients [21]. Certainly, epigenetic alterations in promoter region and the resulting suppression of gene expression both perform in a similar way and in particular cases equivalent to genetic mutations in certain diseases [22].

Concerning *MTHFR* C677T polymorphism, a study was done in Egypt involving 70 women with unexplained pregnancy loss and 136 healthy fertile controls stated that *MTHFR* C677T was not associated with RPL. CT, TT genotypes were increased in cases compared to controls but did not reach statistical significance which was in an agreement with our results [23]. Also other studies concluded that *MTHFR* C677T was not a predictive risk for recurrent pregnancy loss in different ethnic groups [24–26]. In contrast to our *MTHFR* PCR-RFLP results, a study performed in Iran involving one hundred and fifty three females revealed that CT and TT for C677T polymorphism were 18.13% and 13.1%. And all participants who were homozygous for C677T were treated accordingly with anti-coagulant therapy and ended with successful pregnancy [27]. Other studies supported the association of *MTHFR* C677T polymorphism with RPL as well [28, 29].

This raised our attention to a question: what could be the possible other epigenetic causes that might lead to hypermethylation in promoter region of M*THFR* gene among women suffering from RPL irrespective to *MTHFR* C677T polymorphism. We believe that in cases of RPL especially (unexplained RPL), early finding of new prognostic marker as DNA hypermethylation would be of great help to obstetricians for early detection of RPL cases. There are several theories correlating hypermethylation status found among the DNA of patients with unexplained recurrent pregnancy loss. It is proposed that hypermethylation status of *MTHFR* promoter region found among RPL patients favor the direction of epigenetic silencing, leading to a decreased accessibility of methyl groups needed in the epigenetic processes which is crucial for normal fetal development and results in pregnancy loss [19]. Other theory is how the immune system might have different response regarding the acceptance and accommodation of the fetus. Because hereditary spontaneous modifications in DNA methylation have a significant roles in the normal function process of the immune system, for instance in T-cell biology [30]. Changes in DNA methyltransferase (DMT )and ten-eleven translocation( TET) enzyme levels have a role in fluctuation of DNA methylation status as well as hydroxymethylation process [31]. La Rocca and his colleagues [32] done a study which supports this point of view. Epigenetic profile of the genome is reprogrammed dynamically during the early development of the fetus [19].

Also taking insights among surrounding environmental factors especially in our country for example environmental maternal exposure to extreme life events (pollution, excessive passive smoking, heavy load work, lack of healthy diet) especially among low socioeconomic class women earlier or throughout the time of conception may be related with changes in the normal epigenetic mechanisms. Epigenetic mechanisms are believed to arbitrate environmental effects on gene regulation and to moderate susceptibility to certain diseases [33, 34]. Though pregnancy loss is considered to be a psychological letdown because of the loss of the fetus but looking at it from the another point of view, it may be considered fortunate as the abnormal epigenetic load from the maternal side gets lost and not transferred to the next generation. Our study is limited by the fact that we could not analyze fetal tissue as well; results would have been more valid and interesting after comparison of feto-maternal methylation patterns and that it was difficult to collect adequate controls.

## Conclusion

There is a correlation between methylation statuses of *MTHFR* coding region with RPL. This differential methylation pattern observed among RPL cases could possibly be responsible for the inadequate maternal uterine environment. This study highlights the significance of epigenetic mechanisms in pregnancy loss. As methylation process plays an essential role in the fetus programming and the expected wellbeing of the offspring. Recognition of abnormal methylation levels early during gestation period might help in early detection RPL cases. This study is a primarily step for other researches to be done on the topic but preverbal on larger sample size studies before making any solid conclusions and additionally offers a new vision in recognizing the underlying epigenetic markers triggering RPL.

## Data Availability

The datasets generated and/or analyzed during the current study are not publicly available due to patient’s privacy but are available from the corresponding author on reasonable request.

## Abbreviations

AUC: Area under the curve
BMI: Body mass index
CI: Confidence interval
CpG: Cytosine–phosphate–guanine
DMT: DNA methyltransferase
EDTA: Ethylenediaminetetraacetic acid
MTHFR: Methylenetetrahydrofolate reductase
OR: Odds ratio
RFLP: Restriction fragment length polymerase
RPL: Recurrent pregnancy loss
ROC: Receiver operating characteristic
TET: Ten-eleven translocation
